# High-Risk Human Papillomavirus in Patients with Oral Carcinoma and Oral Potentially Malignant Disorders in Serbia—A Pilot Study

**DOI:** 10.3390/medicina59101843

**Published:** 2023-10-17

**Authors:** Anđelija Petrović, Miloš Čanković, Miloš Avramov, Željko D. Popović, Srđa Janković, Slavko Mojsilović

**Affiliations:** 1 Group for Hematology and Stem Cells, Institute for Medical Research, University of Belgrade, 11000 Belgrade, Serbia; andjelija.petrovic@imi.bg.ac.rs; 2 Faculty of Medicine, University of Novi Sad, Hajduk Veljkova 3, 21000 Novi Sad, Serbia; milos.cankovic@mf.uns.ac.rs; 3 Oral Medicine Section, Dentistry Department, Clinic for Dentistry of Vojvodina, Hajduk Veljkova 12, 21000 Novi Sad, Serbia; 4 Department of Biology and Ecology, Faculty of Sciences, University of Novi Sad, Trg Dositeja Obradovića 2, 21000 Novi Sad, Serbia; milos.avramov@dbe.uns.ac.rs (M.A.); zeljko.popovic@dbe.uns.ac.rs (Ž.D.P.); 5 Molecular Diagnostic Laboratory, GenoLab, Kosovska 7, 21000 Novi Sad, Serbia; 6 Division of Immunology, Department of Hematology and Oncology, University Children’s Hospital, Tiršova 10, 11000 Belgrade, Serbia; srdja.jankovic@udk.bg.ac.rs

**Keywords:** human papillomavirus, oral squamous cell carcinoma, oral potentially malignant disorders

## Abstract

*Background and Objectives:* Oral squamous cell carcinoma (OSCC) accounts for about 95% of oral cancers. It represents a serious public health problem due to the high degree of morbidity and mortality, as well as multifactorial etiology. Human papillomavirus (HPV) infection is a well-documented risk factor for oropharyngeal carcinoma, but its role in oral carcinogenesis is still debatable. Our aim was to investigate the differences in the prevalence of high-risk HPV genotypes (HR-HPV) in patients with OSCC and oral potentially malignant disorders (OPMD) from that of healthy subjects. *Materials and Methods:* A total of 90 subjects were included in the cross-sectional study and divided into three groups of 30 patients each: (1) patients with OSCC, (2) patients with OPMD, and (3) healthy subjects. We examined the presence of 12 HR-HPV genotypes in the obtained biological material (oral swabs) using real-time PCR. *Results:* One or more of the 12 tested HR-HPV genotypes were detected in 5/30 patients with OSCC and 2/30 with OPMD, whereas no healthy subjects were positive for any of the tested genotypes. There was a statistically significant difference in nodal involvement between HPV-positive and HPV-negative patients with OSCC. *Conclusions:* Oral HR-HPV was detected in patients with oral premalignant and malignant lesions but not in healthy individuals, suggesting a possible role in oral carcinogenesis. Broad HR-HPV panel testing could increase the sensitivity of risk assessment and screening for OSCC.

## 1. Introduction

Oral squamous cell carcinoma (OSCC) is a subset of head and neck squamous cell carcinoma (HNSCC) and accounts for over 90% of all oral cancers. It is the sixth-most common type of carcinoma, with 400,000 new cases annually worldwide. In addition, its local aggressiveness, late onset of symptoms, and tendency to metastasize early to regional lymph nodes, as well as relatively poor response to treatment, with a five-year survival rate under 50%, make this disease an important global public health problem [1,2,3]. According to the last report, the incidence of cancers of the oral cavity in the Republic of Serbia is 15/100,000 men and 4.96/100,000 women per year [4]. Well-known risk factors include smoking, alcohol, and betel quid chewing, as well as chronic inflammation of any cause [2]. In recent decades, a progressive increase in the incidence of oral cancer has been noticed. It appears to be unrelated to known etiologic factors, affecting non-smokers and patients who do not consume alcohol [5,6]. This so-called “oral cancer of the young” is a matter of debate, and appropriate research models for analyzing this entity are still lacking. Some microorganisms have been documented to contribute to carcinogenesis in certain settings. Along these lines, recent studies have shown that oral microbiota may be a factor in the etiology of precancerous lesions (and thus cancer) and may alter disease severity and response to treatment [7,8,9]. The microenvironment that contributes to tumor growth and invasion may be created by certain specific microbes and microbial communities. Their potential role in this complex process is to destroy the epithelial barrier, damage the tissue, disrupt the local immune response, and even exert genetic toxicity (mutagenesis). In addition, some chemical products of the physiological microbiota are known to react with DNA, potentially promoting malignant cell transformation [10].

An important group of mucosal disorders are oral potentially malignant disorders (OPMD) that may precede OSCC. OPMD is defined as any oral mucosal abnormality that is associated with a statistically significant risk of developing oral cancer [11]. This group of disorders comprises oral leukoplakia (OLK), oral lichen planus (OLP), proliferative verrucous leukoplakia, oral submucous fibrosis, actinic cheilitis, dyskeratosis congenita, oral lichenoid lesions, palatal lesions of reverse cigar smoking, discoid lupus erythematosus, and oral graft-versus-host disease [12].

Human papillomavirus (HPV) is currently the focus of many studies [5,9,13]. In the last decade, the worldwide prevalence of HPV infections has increased rapidly. Established risk factors for HPV infection include immunodeficiency, alcohol, smoking, number of sexual partners, number of oral sex partners, and open-mouth kissing [2,13]. Cancers associated with HPV infection to date include cervical, vulvar, vaginal, penile, anal, urological, and head and neck cancers [3,14,15,16]. A potential role of HPV in the development of OSCC has been debated since 1983 [17]. However, the role of HPV in the progression of OPMD to OSCC is still unclear [18,19]. In general, HPV exhibits squamous epithelial tropism, and basal cells of the epithelium are exposed to viral particles through microabrasions or epithelial injury [20]. Integration of HPV DNA into the host cell genome leads to increased expression of oncoproteins E6 and E7. The activity of these oncoproteins leads to genomic instability, cell cycle arrest, cell proliferation, immortalization, and malignant transformation of HPV-infected cells. In addition, the immunosuppression caused by these oncoproteins prevents immune recognition of HPV-infected and transformed cells [21].

So far, scientists have identified over 200 HPV genotypes and classified them into low-risk (LR) and high-risk (HR) genotypes of inducing oncogenesis. Unlike most studies that examine the already well-known HR-HPV genotypes 16 and 18, we included in our study as many as twelve HR-HPV genotypes associated with carcinogenesis (16, 18, 31, 33, 35, 39, 45, 51, 52, 56, 58, and 59) that have been classified as carcinogenic to humans by the International Agency for Research on Cancer [14].

Given that HR-HPV is recognized as one of the etiological factors not only for OSCC but for OPMD as well, we included in our study patients with a histologically confirmed diagnosis of oral lichen planus and leukoplakia. Our pilot study is one of the few studies conducted on the territory of the Republic of Serbia with the aim of investigating the prevalence of oral HR-HPV infection in OSCC and OPMD patients and comparing it with a healthy control group. Documenting an association between HPV infection and premalignant or malignant lesions of the oral cavity might advance our understanding of the etiology of such lesions and pave the way for improving screening and risk assessment of HPV-associated oral malignancies and possibly for new therapeutic approaches.

## 2. Materials and Methods

### 2.1. Study Population and Sample Collection

This study was conducted as a cross-sectional pilot study at the Clinic for Maxillofacial Surgery of the Clinical Center of Vojvodina and the Clinic for Dentistry of Vojvodina in Novi Sad, Serbia, between December 2018 and April 2022. We enlisted 90 subjects (60 patients and 30 controls). This study was approved by the Ethics Committee of the Clinical Center of Vojvodina (approval No. 00-20/654) and was conducted in full accordance with ethical principles and the Declaration of Helsinki. Each subject signed a written informed consent form prior to enrolment in this study.

The exclusion criteria were respondents younger than eighteen years of age, malignant diseases of other localizations, systemic and autoimmune diseases, acquired immunodeficiency syndrome, acute inflammation of the oral cavity, chronic inflammation of the periodontium, previous treatment for cancer of the oral cavity, current chemotherapy and/or radiotherapy, pregnancy, and breastfeeding.

The first patient group consisted of 30 patients in whom the diagnosis of OSCC was clinically and histopathologically verified. The second group consisted of 30 patients with a clinically and histopathologically verified diagnosis of OPMD, including OLK and OLP. The third group consisted of 30 people without pathological changes in the oral cavity who came to the Clinic for Dentistry of Vojvodina for a regular dental check-up. The subjects were given instructions not to consume food or drinks (except water) and not to smoke for at least 30 min before sampling. Material was sampled using swabs (Sigma Dry Swab, MW914, Sigma-Aldrich, St. Louis, MO, USA) from the lesions (OPMD, OSCC) or buccal mucosa (control subjects). The swabs were immediately transferred into transport medium and stored at −20 °C until extraction. The presence of 12 high-risk HPV genotypes (16, 18, 31, 33, 35, 39, 45, 51, 52, 56, 58, and 59) was detected by real-time quantitative polymerase chain reaction (qPCR).

### 2.2. DNA Extraction and HPV Genotyping

DNA was isolated from clinical specimens using the RealBest DNA-Express CE IVD extraction kit C-8899 (AO Vector-Best, Novosibirsk, Russia; BIORON GmbH, Römerberg, Germany) according to the manufacturer’s instructions. Cells from oral swabs were stripped in a tube with a transport medium. Then, 100 μL of transport medium with cells was transferred into a lysis buffer tube that was incubated at 98 °C for 10 min. When cooled, samples were centrifuged at 8000 rpm for 15 min, and supernatant rich with DNA was used for subsequent PCR reactions.

Genotyping was performed using RealBest DNA HPV HR genotype CE IVD diagnostic kit D-8444 in strip format (AO Vector-Best, RU; BIORON GmbH, Germany) following the manufacturer’s instructions and on CFX96 IVD thermal cycler (BioRad, Hercules, CA, USA). The RealBest DNA HPV HR Genotype assay kit is designed for the differential determination of DNA of HR-HPV types 16, 18, 31, 33, 35, 39, 45, 51, 52, 56, 58, and 59. The genotyping kit analyzes 12 HPV genotypes in 4 separate reactions, each containing 3 HPV genotypes and internal control (IC) or human beta-actin gene in the FAM, HEX, ROX, and Cy5 channels.

For the PCR reaction, 50 μL of DNA samples of either specimen, negative control (NC), or positive control (PC) were pipetted into 4 separate reaction tubes on the PCR strip and left to incubate for 20 min in a thermal cycler protected from light.

The PCR genotyping reaction was performed by applying the following thermal conditions: Stage 1: 50 °C, 2 min; Stage 2: 95 °C, 2 min; Stage 3: 50 cycles of step 1: 94 °C, 10 s, and step 2: 60 °C, 25 s (fluorescence recording).

The PCR run was valid only if all reactions had positive IC signals. For NC reactions, IC signals were present in 3 reactions, while the signal from the human beta-actin gene was absent. In the PC reactions, signals from the IC and all analyzed genotypes were recorded, while for each patient, signals from the beta-actin gene and signals from the 3 IC were monitored. In the absence of a signal from the human beta-actin gene, the reaction was discarded as invalid.

### 2.3. Statistical Analysis

For the statistical processing of data, the commercial statistical package SPSS 23 for Windows was used. For attributive characteristics, the data were shown in the form of absolute and relative numbers and for numerical characteristics, as mean values and measures of variability. ANOVA was applied for significance testing of differences between the three groups for numerical parameters, and Fisher’s exact probability test or the χ^2^ test (with Yates’ correction) was applied for attributive characteristics. The *p*-value < 0.05 was considered statistically significant.

## 3. Results

### 3.1. Demographic Characteristics

Demographic characteristics and habits of patients and control subjects enrolled in this study are presented in Table 1. OSCC patients and controls were age- and gender-matched. OPMD patients were younger than controls and OSCC patients. OPMD was more prevalent in females (2/3), whereas male predominance was seen in OSCC. OSCC patients were predominantly of a lower educational level than OPMD patients and control subjects. Of the known risk factors, the highest proportion of smokers was in the OSCC group, while alcohol consumption was rarely reported in any of the three groups. A family history of cancer was shown not to be a significant risk factor. There were also differences in employment status between participants of different groups, which was in accordance with the groups’ mean age (Table 1).

### 3.2. Prevalence of HR-HPV Genotypes

**Figure 1:**
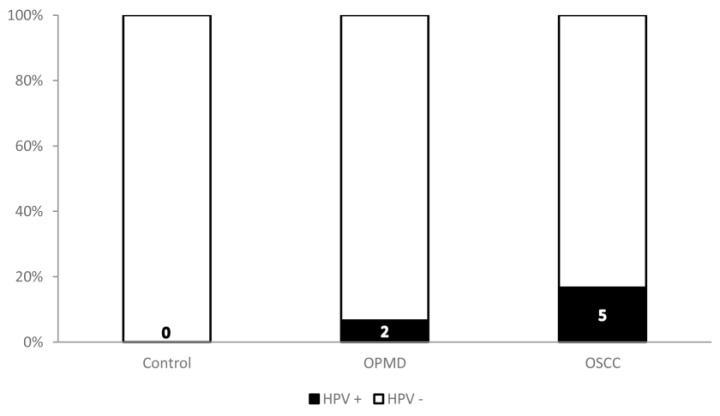
HPV status of the control group, OPMD group, and OSCC group. Digits represent the number of HPV-positive patients per group.

Real-time PCR analysis revealed the presence of HR-HPV DNA in oral swab specimens of some OSCC and OPMD patients but none in the healthy subjects. We found HPV DNA in 5/30 (16.7%) of the OSCC samples and 2/30 (6.7%) of OPMD samples (Figure 1 and Table 2).

Of the 12 high-risk HPV genotypes that were analyzed, we detected the following five types: 33, 39, 51, 52, and 59. The detected HPV genotypes and their distribution among the three groups are shown in Table 2.

### 3.3. HR-HPV and Clinicopathological Characteristics of OSCC

Clinicopathological characteristics of OSCC are presented in Table 3. The most prevalent localization of the tumor was the tongue and floor of the mouth (63.3%). The depth of invasion was almost evenly distributed, with 46.7% of OSCC invading tissue 1 cm or less and 53.3% more than 1 cm beyond the basement membrane. According to the staging of OSCC, most tumors were in the advanced clinical stages (stages III and IV), and regional metastases were present in the lymph nodes of the neck in 19/30 (63.3%) patients. There was a statistically significant difference (*p* = 0.047) in nodal involvement among HPV-positive OSCC (Table 3), showing a higher prevalence of HR-HPV infection (36.3%) in patients without regional metastasis than in those with nodal involvement (5.2). The distribution of the HPV-positive OSCC among other clinicopathological categories was without statistical significance.

Regarding OPMD, four of the 30 cases were OLK with mild dysplasia, and the others were OLP without dysplasia. Two OLP lesions were HPV-positive, which did not show a statistically significant correlation.

## 4. Discussion

Some viruses are associated with cancer development in humans, including Epstein–Barr virus, hepatitis B virus, hepatitis C virus, HPV, human T-cell lymphotropic virus, Kaposi’s sarcoma-associated herpesvirus, and Merkel cell polyomavirus [10,22]. These oncogenic viruses induce changes in cellular functions that ultimately lead to cancer development and account for 10–15% of cancers worldwide [10]. The integration of HPV DNA into the human keratinocyte genome leads to the active expression of the E6 and E7 oncogenes. These two oncogenes are responsible for the inactivation of p53 and Rb pathways and other cellular, genomic, and epigenetic changes that contribute to carcinogenesis [20,21,22]. The active immunosuppression, as well as the secluded lifecycle of HR-HPV that entirely proceeds within the epithelium, without viremia, cell lysis, or inflammation, favor persistent infection and an increased probability of progression toward invasive cancer [20]. This emphasizes the need for early detection of HR-HPV infections.

The involvement of HPV infection in the etiopathogenesis of oral malignancies has been a focus of debate for several decades. Although the impact of HPV has been proven in the development of oropharyngeal cancer, especially tonsil cancer, its definitive association with cancers of the oral cavity is yet to be verified. Analyzing the World Summary Report on HPV and related diseases, one can observe that of all HPV-related cancers (cervical, anal, vulvar, vaginal, penile, oropharyngeal, oral, and laryngeal) cervical cancer is the most common (incidence rate 13.3/100,000), followed by oral (men 5.96 and women 2.28) and oropharyngeal cancer (men 1.79 and women 0.40) [3]. Klozar et al. detected HPV in 70% of patients with oropharyngeal carcinoma (of which 80% were tonsillar carcinoma), while only 20% of oral carcinomas were HPV-positive [23]. In general, among patients with OSCC, the prevalence of HPV is around 25% [24,25,26]. A detailed overview of the prevalence of human papillomavirus in oral cancer worldwide can be found in a systematic review by Katirachi et al. [27].

The geographical component has a significant impact since the highest prevalence of HPV was recorded in Africa and Asia, especially in Chinese studies conducted in regions with a high incidence of OSCC [25]. Katirachi et al. reported in their systematic review a great heterogeneity in the prevalence of HPV-positive OSCC around the world, ranging from 0% to 37% (in Jordan) [27]. In their meta-analysis of the prevalence of HPV in head and neck cancers in European populations, Abogunrin et al. found that the prevalence of HPV infection in oral cancers was slightly lower, although not significantly, for populations in Western Europe than in Eastern Europe (21.3% vs. 34.7%, respectively) [28].

Our study’s population was mostly composed of elderly (median age 65) residents of the Autonomous Province of Vojvodina, Republic of Serbia. Among the usual risk factors, smoking was more ubiquitous in OSCC patients, while alcohol consumption and genetic background did not present as significant risks in our study. Other intriguing findings were differences in education and employment status among the studied groups. Namely, OSCC patients were predominantly retirees of lower educational level. Interestingly, Lins et al., in their socio-demographic study, observed that the advanced stage of OSCC is more common in males (78.4%) with completed primary education (59.1%) and under single marital status (46%) [29]. Although not statistically significant, it is noteworthy that in our study, the majority of cancer patients (56.7%) were single, while most of the healthy subjects had a partner. While this could be just a coincidental finding, some public health specialists point out that people engaged in committed relationships had more self-care, as well as health support from their partner, who tends to perceive the changes in the spouse and alert them [30].

In this cross-sectional pilot study, we analyzed 12 high-risk HPV types in OSCC and OPMD patients, as well as healthy control subjects. There have been few studies on HPV infection in OSCC or OPMD patients in Serbia. Kozomara et al. found 64% of HPV-positive samples in 50 patients with squamous cell carcinoma of the tongue or floor of the mouth [31]. Furthermore, Milovanović et al. have shown a prevalence of HPV of 55.2% in oropharyngeal squamous cell carcinoma patients [32]. Our study confirmed HPV positivity in 5 (16.7%) patients with OSCC and 2 (6.7%) patients with OPMD, while there were no HPV-positive samples among healthy subjects. Although this result is not statistically significant, the presence of HPV among cancer patients and its absence in healthy controls may be taken as a suggestion of a role of HPV in oral carcinogenesis. This relatively low number of HPV-positive OSCC and OPMD patients in our study may be due to the relatively small sample size but also to the predominantly elderly population studied, as HPV-associated head and neck carcinomas are more prevalent in the younger population [33].

However, very similar results were reported by Božić et al., who aimed to determine the major risk factors for the development of OSCC in Serbia. Among 33 patients treated for OSCC and 30 patients with benign lesions, they found 5 (15.2%) and 2 (6.7%) HPV-positive samples, respectively [34]. Investigators from other regions had comparable findings. Yang et al. showed that HPV was present in 3.3% (1/30) of patients in the OSCC group, 4.9% (5/103) in the leukoplakia group, and 3.3% (1/30) in the control group [35]. Also, Cao et al. found no statistically significant difference in the presence of HPV among patients with OSCC (7.1%) and those with premalignant lesions (12.1% of OLP and 2.6% of OLK), which is in accordance with the results of our study [36]. According to research that included 13,089 adults aged 20–69, oral mucosal HPV infection in healthy individuals had a prevalence of 3.5% [37], while some other studies had a prevalence of HR-HPV genotypes in healthy individuals of around 1% [38,39].

Out of the 12 HR-HPV genotypes analyzed in this study, we detected five: 33, 39, 51, 52, and 59, in OSCC patients and 33, 52, and 59 in OPMD patients. Although those genotypes are associated with oral carcinogenesis [27], it is interesting that the most common HR-HPV genotypes, 16 and 18, were not detected in our cohort. This might be attributed to the small number of positive patients, and a larger study could reveal the presence of other HR-HPV genotypes as well. It should also be noted that HPV genotypes 16 and 18 are often the only high-risk genotypes tested, and it is possible that the other HR-HPV genotypes are more prevalent than the literature data suggest. In any case, these results imply that broader testing for HR-HPV genotypes is advisable to increase the sensitivity of risk assessment for OSCC.

A series of molecular, metabolic, and histologic changes accompany the oral cancer progression of OPMD and can be used as indicators of malignant transformation. Oral epithelial dysplasia represents a transitional phase in the malignant transformation of OPMD, as dysplastic OPMDs exhibit a higher risk of progression to OSCC [12]. Some cancer stem cell markers have shown a positive correlation with the degree of dysplasia, including CD44, retinal dehydrogenase 1, prominin-1, and podoplanin [40,41]. In a recent study, Salama et al. demonstrated a correlation between hypoxia-inducible factor 1 alpha and CD44 expression with epithelial dysplasia, showing a higher percentage of immunostained cells in samples with a higher degree of dysplasia [41]. However, there is still insufficient data to incorporate such biomarkers into the clinical decision-making process. Our OPMD study group comprised mostly OLP without dysplasia (26/30) and only four cases of OLK with mild dysplasia, which precluded finding any correlation. It would be interesting for future studies to include more diverse OPMD cases with varying degrees of dysplasia to determine whether HR-HPV status may indicate a potential for OPMD to undergo malignant transformation.

Some research groups have tested the hypothesis that HR-HPV plays a role in carcinogenesis with OPMD background. Syrjänen et al. hypothesized that at a very early stage of carcinogenesis, the ability of epithelial cells to duplicate is increased when apoptotic pathways are blocked by oncogenic proteins E6 and E7 [42]. They studied the risk of HPV infection in patients with OPMD and concluded that it increased nearly four times in oral leukoplakia and oral lichen planus compared with healthy oral mucous membranes. The same authors calculated that HPV infection was thirteen times more prevalent in squamous cell carcinoma of the oropharynx and four times more present in OSCC than in healthy mucous membranes, especially in regard to HPV16. Therefore, the possibility that the lesions facilitate the entry of the virus cannot presently be excluded.

Numerous studies have evaluated HPV status as a prognostic factor for head and neck cancer patients. The prognostic relevance of biologically active HPV infection is clearly established in oropharyngeal squamous cell carcinoma, showing a better prognosis for HPV-infected patients [9]. However, there is no such clear association for OSCC. There are a number of contradictory studies, some in favor of better odds of survival for HPV-positive OSCC patients, others of a worse outcome, and several studies support no effect on patient survival [9,31,32,43]. Since this is a cross-sectional study, we do not have insight into patients’ outcomes. However, it is indicative that in our study, the presence of regional metastases among HPV-positive OSCC patients is significantly lower compared with HPV-negative patients. Even though there are no other significant differences between HPV-positive and HPV-negative patients when comparing other clinicopathological features of cancer, we believe that more in-depth studies are warranted to assess the prognostic significance of HPV infection in OSCC and OPMD patients.

## 5. Conclusions

This pilot study demonstrated a difference in HR-HPV prevalence between OSCC patients and healthy individuals that merits further investigations, on a larger cohort, to properly assess the role of these oncogenic viruses in the process of carcinogenesis at this localization. If such a link is confirmed, the analysis of HR-HPV could, in the future, become part of a panel of biomarkers that would increase the sensitivity of cancer screening by detecting pathological alterations in biological material (smear of the oral cavity, saliva) and combining it with molecular-level data. Early diagnosis of oral cancer is crucial for improving patient survival rates and the detection of inapparent HR-HPV infection in otherwise healthy individuals or, in patients with OPMD, may forewarn a higher risk of malignant transformation. Moreover, the prevention of such infections, e.g., through the use of vaccines that have already demonstrated effectiveness in preventing other HPV-associated carcinomas, could offer an additional layer of prevention of this life-threatening disease.

## Figures and Tables

**Table 1:** Characteristics of the control group, OPMD group, and OSCC group.

Characteristic		Control *n* = 30	OPMD *n* = 30	OSCC *n* = 30	*p*
Mean age (range)		66.03 (45–87)	56.73 (31–80)	66.10 (47–86)	0.04
Gender (%)	Female	11 (36.7)	20 (66.7)	11 (36.7)	0.02
	Male	19 (63.3)	10 (33.3)	19 (63.3)	
Education (%)	Elementary school	7 (23.3)	7 (23.3)	12 (40.0)	0.02
	High school	15 (50.0)	9 (30.0)	15 (50.0)	
	University	8 (26.7)	14 (46.7)	3 (10.0)	
Employment (%)	Unemployed	4 (13.3)	3 (10.0)	5 (16.7)	0.04
	Employed	6 (20.0)	14 (46.7)	4 (13.3)	
	Retired	20 (66.7)	13 (43.3)	21 (70.0)	
Partner (%)	With partner	21 (70.0)	18 (60.0)	13 (43.3)	0.10
	Without partner	9 (30.0)	12 (40.0)	17 (56.7)	
Body mass index (%)	≤18.5	1 (3.3)	0 (0.0)	1 (3.3)	0.60
	18.51–25	10 (33.3)	9 (30.0)	13 (43.3)	
	≥25.01	19 (63.3)	21 (70.0)	16 (53.3)	
Smoking (%)		8 (26.7)	10 (33.3)	19 (63.3)	0.01
Alcohol (%)		4 (13.3)	3 (10.0)	8 (26.7)	0.10
Family history of cancer (%)		15 (50.0)	14 (46.7)	7 (23.3)	0.07

**Table 2:** Distribution and genotypes of high-risk HPV of the control group, OPMD group, and OSCC group.

Group	HPV-Positive (%)	HPV Types	HPV-Negative (%)	*p*
OSCC	5/30 (16.7)	33, 39, 51, 52, 59	25/30 (83.3)	0.053
OPMD	2/30 (6.7)	33, 52, 59	28/30 (93.3)	
Control	0/30 (0.0)	N/A	30/30 (100.0)	

**Table 3:** Clinicopathological characteristics and HPV status of OSCC patients.

Tumor Characteristic		Total Number of Tumors	Number of HPV-Positive Tumors	*p*
Localization (%)	Tongue and floor of the mouth	19/30 (63.3)	3/19 (15.8)	>0.999
	Gingiva	4/30 (13.3)	1/4 (25.0)	
	Lips	4/30 (13.3)	1/4 (25.0)	
	Buccal	3/30 (10.0)	0/3 (0.0)	
Depth of invasion (%)	≤1	14/30 (46.7)	3/14 (21.4)	>0.999
	>1	16/30 (53.3)	2/16 (12.5)	
Stage (%)	I	3/30 (10.0)	1/3 (33.3)	>0.999
	II	5/30 (16.7)	2/5 (40.0)	
	III	12/30 (40.0)	1/12(8.3)	
	IV	10/30 (33.3)	1/10 (10.0)	
Regional metastasis (%)	No	11/30 (36.7)	4/11 (36.3)	0.047
	Yes	19/30 (63.3)	1/19 (5.2)	

## Data Availability

The data presented in this study are available upon request from the corresponding author. The data are not publicly available due to privacy.
